# Research on the Directional Adaptability of a Self-Adaptive Energy Harvester

**DOI:** 10.3390/s23115106

**Published:** 2023-05-26

**Authors:** Minglei Han, Xu Yang, Shimin Guo

**Affiliations:** Key Laboratory of CNC Equipment Reliability, Ministry of Education, School of Mechanical and Aerospace Engineering, Jilin University, Changchun 130022, China

**Keywords:** directional self-adaptive energy harvester, directional adaptability, three-dimensional excitation, energy harvesting workspace, evaluation method

## Abstract

With the continuous development of wireless sensor networks (WSNs), multi-directional energy harvesting technology has received widespread attention from scholars. In order to evaluate the performance of multi-directional energy harvesters, this paper uses a directional self-adaptive piezoelectric energy harvester (DSPEH) as an example, defines the direction of the excitation in three-dimensional space, and studies the influence of excitations on the key parameters of the DSPEH. The rolling angle and pitch angle are used to define complex excitations in three-dimensional space, and the dynamic response of the excitation changes in a single direction and multiple directions is discussed. It is noteworthy that this work presents the concept of “Energy Harvesting Workspace” to describe the working ability of a multi-directional energy harvesting system. The workspace is expressed by the excitation angle and voltage amplitude, and energy harvesting performance is evaluated by the volume-wrapping method and area-covering method. The DSPEH exhibits good directional adaptability in two-dimensional space (rolling direction); in particular, when the mass eccentricity coefficient is *r* = 0 mm, 100% of the workspace in two-dimensional space is obtained. The total workspace in three-dimensional space depends entirely on the energy output in the pitch direction.

## 1. Introduction

The wireless sensor network (WSN) is a revolution in the field of information sensing and harvesting, and it has introduced profound impacts on human production and life [1,2]. WSN consists of tens of thousands of sensor nodes, each of which includes a sensing module, an information processing module, a wireless communication module, a power management module, and a power supply module, where the power supply module supplies power to the other modules and plays a very important role in WSNs [3,4]. In order to provide clean and sustainable electrical energy for WSNs, researchers use various electromechanical conversion mechanisms, such as piezoelectric [5,6,7], electromagnetic [8,9,10], electrostatic [11,12,13], thermoelectric [14,15,16], and triboelectric [17,18,19] mechanisms to convert the vibration energy near the working environment of WSNs into electrical energy. Piezoelectric energy harvesting technology uses the direct piezoelectric effect of piezoelectric materials to realize the conversion of mechanical energy to electrical energy and has high electromechanical conversion efficiency, which has been widely researched by scholars [20,21].

Generally, the traditional piezoelectric energy harvester (PEH) is designed as a cantilever beam structure with a tip mass. When the external excitation direction is consistent with the normal direction of the cantilever beam, the vibration energy in the environment can be efficiently harvested; when the external excitation direction deviates from the normal direction of the cantilever beam, the harvesting efficiency of the traditional PEH will be significantly reduced. However, the environmental vibrations around WSNs are relatively complex, and the vibration direction is intrinsically random and time-varying. To date, various multi-directional schemes have been proposed to achieve relatively efficient energy harvesting in multiple directions, such as series [22,23], arrays [24,25,26], multi-modal cantilever beams [27,28,29], magnetic/mechanical coupling [30,31,32,33,34,35,36], nonlinear systems [37,38,39], etc. However, the power density of these schemes is not high in practical applications. Furthermore, the complex structures in this series, array, and magnetic/mechanical coupling scheme are not conducive to the demand for WSN miniaturization. Therefore, various special-shaped cantilever beams with simple structures have been developed for multi-directional vibrations [40,41,42,43,44,45,46,47,48]. Unfortunately, these special-shaped cantilever beams cannot achieve efficient energy harvesting in all directions.

On the basis of previous research, scholars have developed some kinds of multi-DOF structures with single cantilever beams to realize directional self-adaptive energy harvesting. Zhao et al. [36] developed a directional self-tuning energy harvester that included a spring–mass system, primary beam, rotary arm, host frame, and base plate, which can harvest two-dimensional vibration energy with high efficiency. Han et al. [49] explored a novel mosquito-inspired piezoelectric energy harvester with a single cantilever beam. The cantilever beam can calibrate its position in any direction in two-dimensional space, realizing self-adaptive and multi-directional energy harvesting, and the average voltage increased by 30% compared with the conventional PEH. Inspired by the vibrational stabilization of hummingbirds, Wang et al. [50] proposed an omnidirectional PEH with autonomous direction regulation capability. Thanks to the large dynamic deformation of the cantilever beam, the harvester realized a voltage output of 52 V in any direction in two-dimensional space.

In summary, in the field of piezoelectric energy harvesting, specially shaped cantilever beams, series or array cantilever beams, and single cantilever beams with multi-DOF can be designed to achieve multi-directional energy harvesting. However, most reports focus on vibrations in two-dimensional space, while external excitation may change in three-dimensional space. In our previously proposed directional self-adaptive piezoelectric energy harvester (DSPEH) [49], we only studied the excitation in two-dimensional space. So far, there is no precise description and definition of the excitation direction in the existing literature, let alone a unified method to evaluate the performance of three-dimensional energy harvesting. This paper takes DSPEH as an example, presents the definition of three-dimensional excitation, puts forward the concept of “Energy Harvesting Workspace”, and proposes an evaluation method for multi-directional energy harvesting, providing a reference for multi-directional energy harvesting technology. The paper is organized as follows. Section 2 puts forward the definition of three-dimensional excitations. Section 3 reveals the variation law of system parameters under three-dimensional excitation. Section 4 presents the physical prototype and experimental setup. Section 5 studies the energy harvesting performance of the system under three-dimensional excitation. Thereafter, the evaluation method of energy harvesting under three-dimensional excitation is given. Section 6 draws conclusions.

## 2. DSPEH and Three-Dimensional Excitation

### 2.1. Design Scheme of the DSPEH

**Figure 1:**
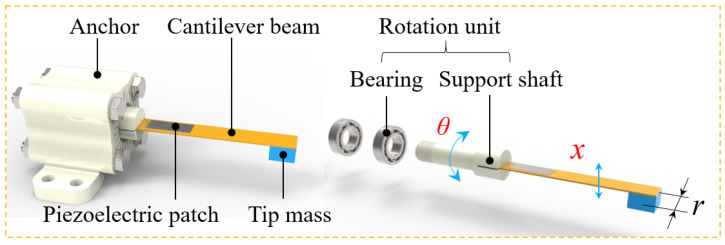
Structure diagram of the DSPEH.

The structure of the proposed DSPEH is shown in Figure 1, which is composed of a cantilever beam, a tip mass, a piezoelectric patch, a rotation unit, and an anchor. The cantilever beam with the piezoelectric patch and the tip mass is installed on the support shaft, and the support shaft is mounted on the anchor using two bearings. Due to the presence of the rotation unit consisting of a support shaft and two bearings, the cantilever beam has two DOFs, and the cantilever can vibrate in its normal direction (*x* direction) and rotate in its axial direction (*θ* direction).

Compared with the conventional PEH, the DSPEH has additional rotational DOFs, which can drive the rotation of the cantilever beam in order to track the direction of the external excitation. When there is an initial angle, *θ*_0_, between the normal direction of the cantilever beam and the excitation direction, the vibrating cantilever beam is in a state of force imbalance. Under the action of the eccentric gravity of the tip mass and vibrating centrifugal force, the cantilever beam begins to rotate to reconstruct the stable state. When the cantilever beam reaches a force equilibrium state, it begins to generate stable vibrations at a certain position without rotation. This adjustment process without any manual intervention or power supply is called a self-adaptive rotation mechanism, which enables the cantilever beam to achieve multi-directional energy harvesting.

### 2.2. Definition of the Three-Dimensional Excitation

**Figure 2:**
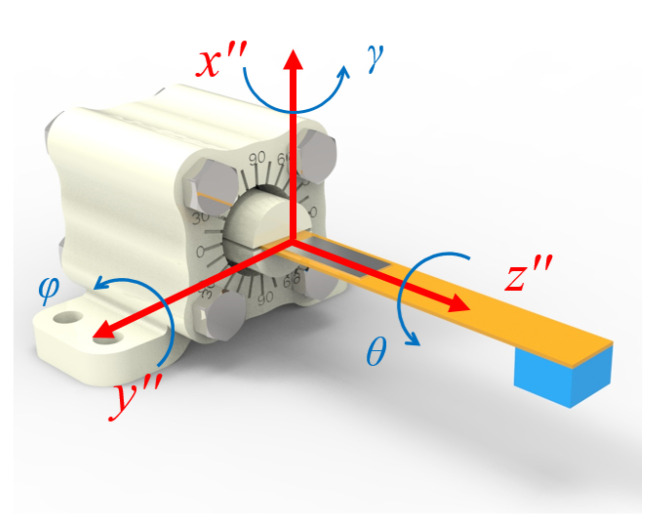
Definition of the three-dimensional excitation.

As shown in Figure 2, in order to describe the direction of the external excitation in coordinate system *x*″-*y*″-*z*″, we use the RPY angle representation method to define three angles: *θ*, *φ*, and *γ*. Here, rolling angle *θ* represents the angle of the external excitation rotating along the *z*″ axis; pitch angle *φ* represents the angle of the external excitation rotating along *y*″ axis; yaw angle *γ* represents the angle of the external excitation rotating along *x*″ axis. The three-dimensional excitation direction is determined by (*θ*, *φ*, and *γ*).

**Figure 3:**
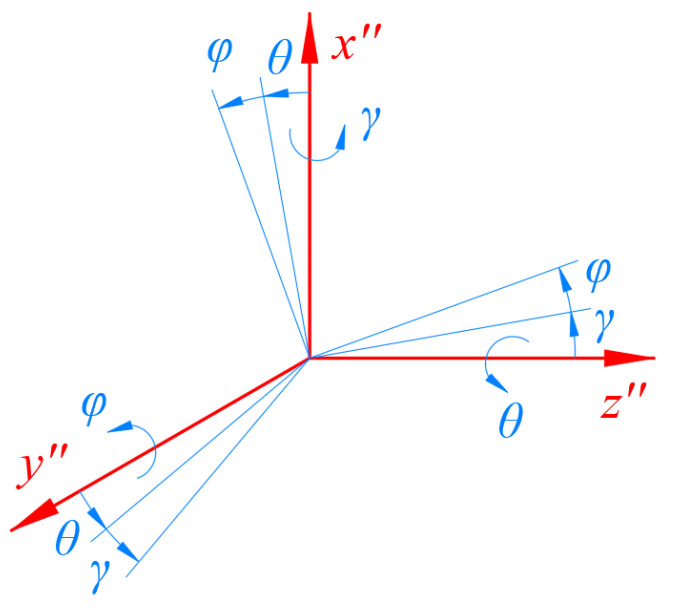
Schematic diagram of the RPY angle’s representation.

The direction of the external excitation can be described as follows: the external excitation first rotates *θ* angle around the *z*″ axis; then, it rotates *φ* angle around the *y*″ axis and finally rotates *γ* angle around the *x*″ axis, as shown in Figure 3. The corresponding rotation transformation formula is shown as follows:

(1) $$\begin{array}{cc} R & =Rot(z,\theta )Rot(y,\varphi )Rot(x,\gamma )\\ & =\left[\begin{array}{ccc} c\theta & -s\theta & 0\\ s\theta & c\theta & 0\\ 0 & 0 & 1 \end{array}\right]\left[\begin{array}{ccc} c\varphi & 0 & s\varphi \\ 0 & 1 & 0\\ -s\varphi & 0 & c\varphi \end{array}\right]\left[\begin{array}{ccc} 1 & 0 & 0\\ 0 & c\gamma & -s\gamma \\ 0 & s\gamma & c\gamma \end{array}\right]\\ & =\left[\begin{array}{ccc} c\theta c\varphi & -s\theta c\gamma +c\theta s\varphi s\gamma & s\theta s\gamma +c\theta s\varphi c\gamma \\ s\theta c\varphi & c\theta c\gamma +s\theta s\varphi s\gamma & -c\theta s\gamma +s\theta s\varphi c\gamma \\ -s\varphi & c\varphi s\gamma & c\varphi c\gamma \end{array}\right] \end{array}$$

where *cφ* = cos*φ* and *sφ* = sin*φ*.

**Figure 4:**
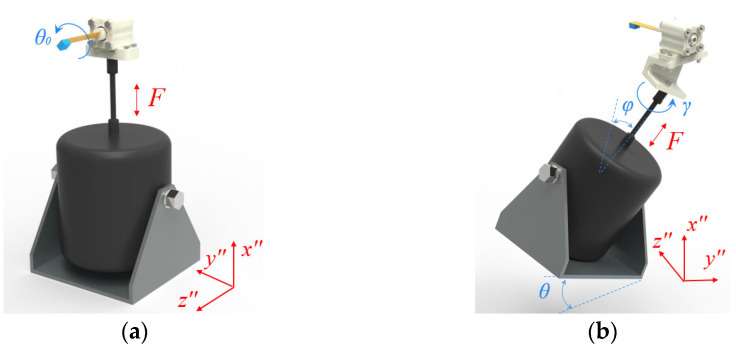
Installation diagram of the energy harvester: (**a**) installation diagram in one direction; (**b**) installation diagram in three directions.

Compared to the research in reference [49], which only discusses the rolling angle along the *z*” axis by assuming an initial angle, *θ*_0_, between the normal direction of the cantilever beam and the excitation direction, this paper adds two angles (*φ* and *γ*) to describe the direction of the external excitation, as shown in Figure 4. The included angles, *θ* and *φ*, are achieved by adjusting the installation angle of the exciter, and the included angle, *γ*, is achieved by adjusting the installation angle of the energy harvester. It should be noted that since the energy harvester is always installed in a certain position, the directional changes with respect to the external excitation in the *x*” axis will not affect the working state of the energy harvester. Therefore, only included angles *θ* and *φ* can be used to determine the excitation direction.

## 3. The Variation Law of System Parameters under Three-Dimensional Excitation

When the external excitation changes in the three-dimensional space, the external excitation and tip mass will exert an axial load on the cantilever beam under the action of the included angle, thereby affecting the natural frequency of the cantilever beam. Additionally, the eccentric gravity of the tip mass will also correspondingly increase the resistance moment on the rotation’s DOF. Therefore, it is necessary to analyze the parameter changes with respect to the cantilever beam under complex excitations in order to correct the relevant parameters of the differential motion equation.

According to Equation (1), based on the coordinate transformation matrix, excitation force *F* acting on the cantilever beam can be decomposed into

(2) $$\left\{ \begin{array}{l} F_{x}=F\cos\theta \cos\varphi \\ F_{y}=F\sin\theta \cos\varphi \\ F_{z}=-F\sin\varphi \end{array}\right.$$

where *F_x_* represents the component of the excitation in the normal direction of the cantilever beam, which can cause the vibration of the cantilever beam; *F_y_* represents the component of the excitation in the tangent direction of the cantilever beam, which can cause the rotation of the cantilever beam; *F_z_* represents the component of the excitation along the *z*″ axis of the cantilever beam, which generates a pre-tension or pre-pressure on the cantilever beam.

### 3.1. Influence of the Three-Dimensional Excitation on the Natural Frequency of the Cantilever Beam

The DSPEH in this paper adopts a cantilever beam structure with a tip mass. Although it has an additional rotation DOF, it still belongs to the ordinary cantilever beam’s structure. Therefore, this paper adopts the same method as ordinary cantilever beams to establish a distributed parameter model, solve the changes in the natural frequency of the system when the excitation direction changes, and optimize the lumped parameter model under harmonic excitations in order to more accurately describe the motion state of the system.

**Figure 5:**
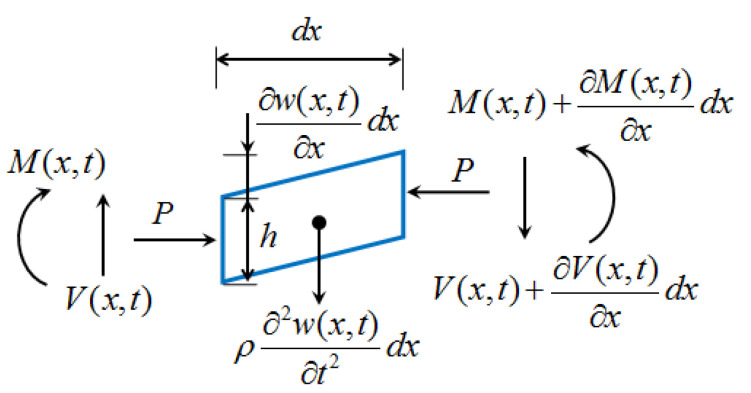
Microelement stress diagram.

Due to the large length-to-thickness ratio of the cantilever beam and the fact that the cantilever beam has rotational DOF in DSPEH, the influence of the shear deformation and rotational inertia of the cantilever beam on the system can be ignored. Therefore, this configuration conforms to the Euler–Bernoulli beam theory. Here, it is assumed that the length of the cantilever beam is *L*, the elastic modulus is *E*, the cross-section of the cantilever beam is equal, the moment of inertia of the cross-section is *I*, and the mass of the cantilever beam per unit length is *ρ*. The lateral vibration displacement of any point on the cantilever beam at time *t* is represented by function *w*(*x*, *t*). When the pre-tension or pre-pressure applied to the cantilever beam is *P* (*P* = *F_z_*), any microelement on the cantilever beam can be taken and its stress is analyzed, as shown in Figure 5.

According to the moment balance and force balance equations of the microelement, we can obtain

(3) $$\left\{ \begin{array}{l} \frac{\partial M(x,t)}{\partial x}dx+P\frac{\partial w(x,t)}{\partial x}dx-V(x,t)dx-\frac{\partial V(x,t)}{\partial x}dxdx=0\\ \frac{\partial V(x,t)}{\partial x}dx+\rho \frac{\partial^{2}w(x,t)}{\partial t^{2}}dx=0 \end{array}\right.$$

Ignoring the second-order small quantity in the first equation of Equation (3), the partial derivative of *x* at both ends of the equation is obtained.

(4) $$\frac{\partial^{2}M(x,t)}{\partial x^{2}}+P\frac{\partial^{2}w(x,t)}{\partial x^{2}}-\frac{\partial V(x,t)}{\partial x}=0$$

Accordingly, the second equation in Equations (3) and (4) can be changed into

(5) $$\frac{\partial^{2}M(x,t)}{\partial x^{2}}+P\frac{\partial^{2}w(x,t)}{\partial x^{2}}+\rho \frac{\partial^{2}w(x,t)}{\partial t^{2}}=0$$

Regardless of geometric nonlinearity, it can be approximately considered that

(6) $$M(x,t)=EI\frac{\partial^{2}w(x,t)}{\partial x^{2}}$$

Then, Equation (6) is substituted into Equation (5).

(7) $$EI\frac{\partial^{4}w(x,t)}{\partial x^{4}}+P\frac{\partial^{2}w(x,t)}{\partial x^{2}}+\rho \frac{\partial^{2}w(x,t)}{\partial t^{2}}=0$$

The time and space functions in Equation (7) are separated and solved by the method of separating variables, assuming that

(8) w(x,t)=ϕ(x)η(t)

Equation (8) is then substituted into Equation (7).

(9) $$EI\frac{d^{4}\phi (x)}{dx^{4}}\eta (t)+P\frac{d^{2}\phi (x)}{dx^{2}}\eta (t)+\rho \frac{d^{2}\eta (t)}{dt^{2}}\phi (x)=0$$

Equation (9) can be written as follows.

(10) $$EI\frac{1}{\phi (x)}\frac{d^{4}\phi (x)}{dx^{4}}+P\frac{1}{\phi (x)}\frac{d^{2}\phi (x)}{dx^{2}}=-\rho \frac{1}{\eta (t)}\frac{d^{2}\eta (t)}{dt^{2}}$$

The left side of Equation (10) depends only on *x*, and the right side depends only on *t*. Since *x* and *t* are independent variables, the standard parameters in the method of separating variables stipulate that both sides of Equation (10) must be equal to the same constant *υ*.

(11) $$EI\frac{1}{\phi (x)}\frac{d^{4}\phi (x)}{dx^{4}}+P\frac{1}{\phi (x)}\frac{d^{2}\phi (x)}{dx^{2}}=-\rho \frac{1}{\eta (t)}\frac{d^{2}\eta (t)}{dt^{2}}=\upsilon =\rho \omega^{2}$$

Equation (11) can be decomposed into two independent equations.

(12) $$EI\frac{d^{4}\phi (x)}{dx^{4}}+P\frac{d^{2}\phi (x)}{dx^{2}}-\rho \omega^{2}\phi (x)=0$$

(13) $$\frac{d^{2}\eta (t)}{dt^{2}}+\omega^{2}\eta (t)=0$$

The general solution of Equation (12) can be expressed as

(14) $$\phi (x)=Ge^{nx}$$

Substituting Equation (14) into Equation (12) yields

(15) $$n^{4}+\frac{P}{EI}n^{2}-\frac{\rho \omega^{2}}{EI}=0$$

The four solutions of the above equation are

(16) $$n_{1,2}=\pm \alpha_{1},\;n_{3,4}=\pm i\alpha_{2}$$

where

$$\alpha_{1}=\sqrt{\sqrt{\frac{\rho \omega^{2}}{EI}+{\left(\frac{P}{2EI}\right)}^{2}}-\frac{P}{2EI}},\;\alpha_{2}=\sqrt{\sqrt{\frac{\rho \omega^{2}}{EI}+{\left(\frac{P}{2EI}\right)}^{2}}+\frac{P}{2EI}}$$

The four solutions are substituted into Equation (14), and they are replaced with trigonometric and hyperbolic functions, yielding

(17) $$\phi (x)=A\cosh\left(\alpha_{1}x\right)+B\sinh\left(\alpha_{1}x\right)+C\cos\left(\alpha_{2}x\right)+D\sin\left(\alpha_{2}x\right)$$

where *A*, *B*, *C*, and *D*; and *α*_1_ and *α*_2_ are determined by boundary conditions.

Equation (13) is a single DOF vibration differential equation, and the general solution can be expressed as

(18) η(t)=Hsin(ωt+ψ)

where *H* and *ψ* are determined by initial conditions.

For a cantilever beam clamped at *x* = 0 and free at *x* = *L* with a tip mass, the boundary conditions of the vibration equation are

(19) $${w(x,t)|}_{x=0}=0$$

(20) $${\frac{\partial w(x,t)}{\partial x}|}_{x=0}=0$$

(21) $${\left[EI\frac{\partial^{2}w(x,t)}{\partial x^{2}}+I_{t}\frac{\partial^{3}w(x,t)}{\partial t^{2}\partial x}\right]}_{x=L}=0$$

(22) $${\left[EI\frac{\partial^{3}w(x,t)}{\partial x^{3}}+P\frac{\partial w(x,t)}{\partial x}-m_{t}\frac{\partial^{2}w(x,t)}{\partial t^{2}}\right]}_{x=L}=0$$

where Equations (19) and (20) are the geometric boundary conditions at *x* = 0, and Equations (21) and (22) are the geometric boundary conditions at *x* = *L*. Here, *m_t_* is the mass of the tip mass, and *I_t_* is the mass inertia moment of the tip mass at *x* = *L*.

Substituting Equations (8), (17), and (18) into Equations (19) and (20) yields

(23) $$\{\begin{array}{c} A+C=0\\ \alpha_{1}B+\alpha_{2}D=0 \end{array}$$

Substituting Equations (8), (17), (18), and (23) into Equations (21) and (22) yields

(24) $$\left[\begin{array}{cc} a_{11} & a_{12}\\ a_{21} & a_{22} \end{array}\right]\left[\begin{array}{c} A\\ B \end{array}\right]=0$$

where

$$\begin{array}{c} a_{11}=\alpha_{1}{}^{2}\cosh\left(\alpha_{1}L\right)+\alpha_{2}{}^{2}\cos\left(\alpha_{2}L\right)-\frac{I_{t}\omega^{2}}{EI}\left[\alpha_{1}\sinh\left(\alpha_{1}L\right)+\alpha_{2}\sin\left(\alpha_{2}L\right)\right]\\ a_{12}=\alpha_{1}{}^{2}\sinh\left(\alpha_{1}L\right)+\alpha_{1}\alpha_{2}\sin\left(\alpha_{2}L\right)-\frac{I_{t}\omega^{2}}{EI}\left[\alpha_{1}\cosh\left(\alpha_{1}L\right)-\alpha_{1}\cos\left(\alpha_{2}L\right)\right]\\ a_{21}=\alpha_{1}{}^{3}\sinh\left(\alpha_{1}L\right)-\alpha_{2}{}^{3}\sin\left(\alpha_{2}L\right)+\frac{M_{t}\omega^{2}}{EI}\left[\cosh\left(\alpha_{1}L\right)-\cos\left(\alpha_{2}L\right)\right]\\ \;\;\;\;\;\;\;\;\;\;\;+\frac{P}{EI}\left[\alpha_{1}\sinh\left(\alpha_{1}L\right)+\alpha_{2}\sin\left(\alpha_{2}L\right)\right]\\ a_{22}=\alpha_{1}{}^{3}\cosh\left(\alpha_{1}L\right)+\alpha_{1}\alpha_{2}{}^{2}\cos\left(\alpha_{2}L\right)+\frac{M_{t}\omega^{2}}{EI}\left[\sinh\left(\alpha_{1}L\right)-\frac{\alpha_{1}}{\alpha_{2}}\sin\left(\alpha_{2}L\right)\right]\\ \;\;\;\;\;\;\;\;\;\;\;+\frac{P}{EI}\left[\alpha_{1}\cosh\left(\alpha_{1}L\right)-\alpha_{1}\cos\left(\alpha_{2}L\right)\right] \end{array}$$

In order to obtain the non-zero solution of the above equation system, the coefficient matrix of the equation must be singular; that is, the following is obtained.

(25) $$a_{11}a_{22}-a_{12}a_{21}=0$$

**Figure 6:**
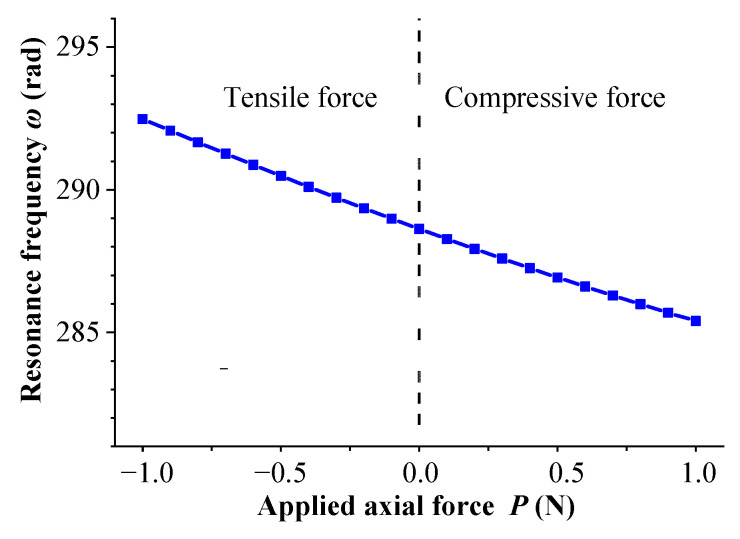
The corresponding curve of natural frequency and axial force.

According to the above equation, the relationship between the natural frequency of cantilever beam *ω* and axial force *P* can be obtained via MATLAB numerical simulations using the Newton Iteration, as shown in Figure 6. From the figure, it can be observed that when the axial force changes within the range of −1.0~+1.0 N, the natural frequency is approximately linearly related to the axial force. When the cantilever beam is stretched, the natural frequency increases with an increase in axial force; when the cantilever beam is compressed, the natural frequency decreases with an increase in axial force.

**Figure 7:**
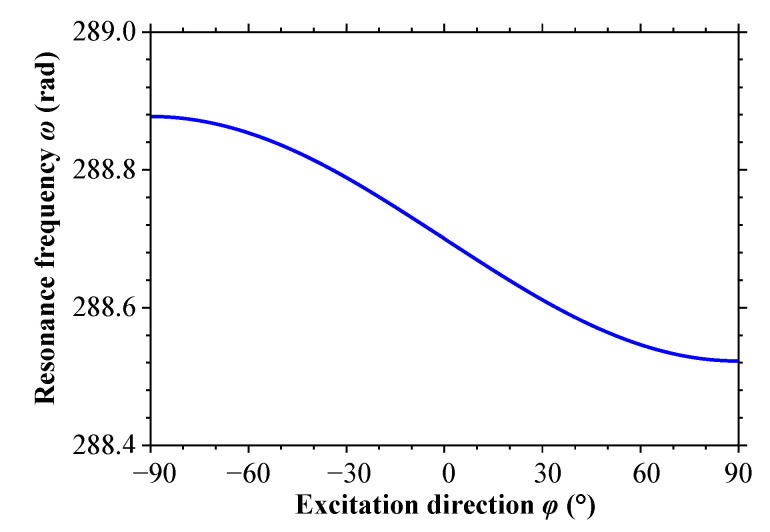
The variation curve of the natural frequency with excitation direction.

The natural frequency of the cantilever beam is related to the axial force. According to Equation (2), the axial force is related to the magnitude of external excitations and pitch angle *φ*, and it is independent of rolling angle *θ*. Figure 7 shows the variation of the natural frequency of the cantilever beam with respect to pitch angle *φ* when the excitation force is *F* = −0.05 N. From the figure, it can be observed that within the range of 0~+90°, the natural frequency of the cantilever beam decreases as pitch angle *φ* increases; within the range of −90~0°, the natural frequency increases as pitch angle *φ* increases.

### 3.2. Influence of the Three-Dimensional Excitation on the Rotational Resistance Moment

When external excitation changes in the rolling and pitch directions, the eccentric gravity of the tip mass will generate a resistance moment, *T_C_*, that acts on the *z*” axis, which will affect the steady-state vibration position and output voltage amplitude of the harvester. This resistance moment can also be obtained using the coordinate transformation matrix.

(26) $$T_{C}=mgr\cos\varphi \sin\theta$$

**Figure 8:**
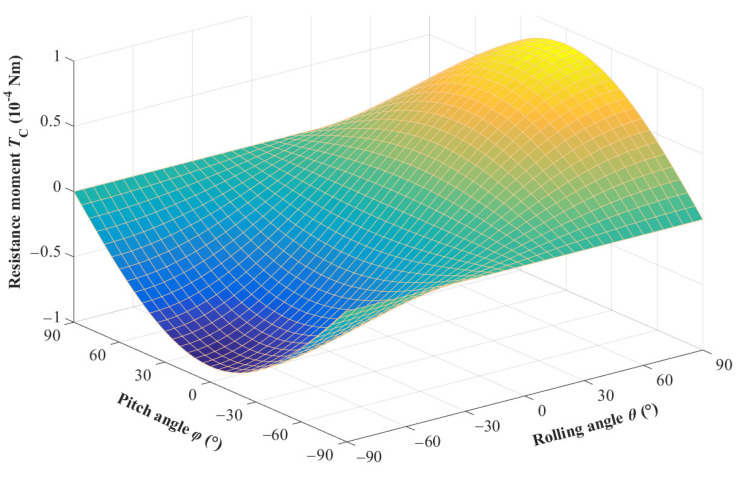
The variation curve of the resistance moment with respect to the excitation direction.

The variation of resistance moment *T_C_* with respect to the excitation direction is shown in Figure 8. From the figure, it can be seen that the resistance moment increases with an increase in rolling angle *θ* and decreases with an increase in pitch angle *φ* within the range of −90~+90°. However, when rolling angle *θ* is zero, the change in pitch angle does not affect the change in resistance moment, so the key factor affecting the resistance moment is rolling angle *θ*.

Based on the above analysis, the dynamic equation of the DSPEH can be expressed as

(27) $$\{\begin{array}{l} m\ddot{x}+c_{1}\dot{x}+kx-m\left(x+r\right){\dot{\theta }}^{2}+mg\left(1-\cos\theta \right)+\alpha V=F\cos\omega^{\prime }t\cos\varphi \cos\theta \\ \begin{array}{l} m{\left(x+r\right)}^{2}\ddot{\theta }+c_{2}\dot{\theta }+2m\left(x+r\right)\dot{x}\dot{\theta }+mg\left(x+r\right)\sin\theta =F\left(x+r\right)\cos\omega^{\prime }t\cos\varphi \sin\theta +mgr\cos\varphi \sin\theta \\ C_{P}\dot{V}-\alpha \dot{x}+\frac{V}{R}=0 \end{array} \end{array}$$

where *m* is the equivalent mass; *c*_1_ is the viscous damping coefficient in the *x* direction; *k* represents the equivalent stiffness; *g* is the gravitational acceleration; *r* is the mass eccentricity coefficient, which represents the distance of the equivalent mass from the center of the cantilever beam, as shown in Figure 1; *α* represents the equivalent electromechanical coupling coefficient; *V* represents the output voltage of the piezoelectric patch; *F* represents the external excitation force; *ω’* represents the frequency of the external excitation; *c*_2_ is the viscous damping coefficient in *θ* direction; *C_P_* represents the equivalent capacitance of the piezoelectric patch; *R* represents the equivalent resistance of the energy harvesting circuit.

## 4. Prototype and Experimental Setup

**Figure 9:**
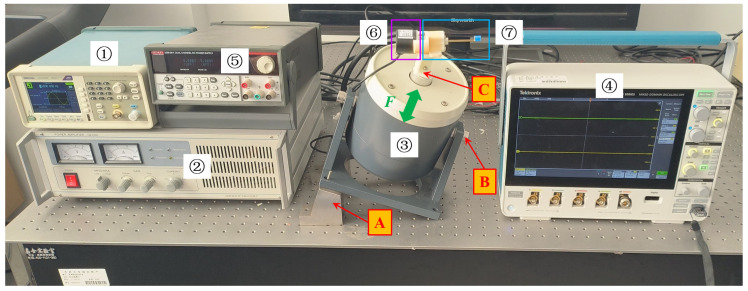
Experimental device diagram of the DSPEH: ① signal generator; ② power amplifier; ③ electromagnetic exciter; ④ oscilloscope; ⑤ DC power supply; ⑥ angle sensor; ⑦ DSPEH.

As shown in Figure 9, the experimental device includes a signal generator, a power amplifier, an electromagnetic exciter, an oscilloscope, an angle sensor, and a DC power supply. The signal generator is used to send out a sine signal, and the power amplifier amplifies and transmits the sine signal to the electromagnetic exciter. The electromagnetic exciter generates a three-dimensional force, *F*, acting on the DSPEH, which is installed on the electromagnetic exciter and outputs the voltage signals of the piezoelectric patch to the oscilloscope. The rotation of the cantilever beam is monitored by the angle sensor and transmitted to the oscilloscope, and the DC power supply is used to supply power to the angle sensor. During the experiment, when rolling angle *θ* or pitch angle *φ* changes separately, adjustment nut B is used on the exciter to adjust the parameters to the required angle, and assembly fixture C is used to ensure the horizontal installation position of the DSPEH. When rolling angle *θ* and pitch angle *φ* change simultaneously, the included angle is adjusted by adjusting fixture A and adjusting nut B, respectively; moreover, fixture C is used to ensure the horizontal installation position of the DSPEH. Rolling angle *θ* and pitch angle *φ* can be adjusted within the range of ±90°. The parameters of the DSPEH are shown in Table 1.

Piezoelectric materials have a critical impact on electromechanical energy conversion. The most common piezoelectric materials include PZT, PVDF, MFC, and so on. Although PZT is widely used for generating electrical energy, its inherent brittleness makes it safe to use only within a certain stress–strain range. PVDF is a piezoelectric polymer with better flexibility than PZT, and it can withstand greater strain and is conducive to generating more electrical energy. Although MFC has good comprehensive performance, the current market price of MFC is tens or hundreds of times higher than that of PZT and PVDF. Therefore, considering the large strain of the cantilever beam and the cost of the experimental system, PVDF is chosen as the piezoelectric material in this paper.

## 5. Results and Discussion

### 5.1. The Output Characteristics of the DSPEH When the Excitation Changes in the Rolling Direction

**Figure 10:**
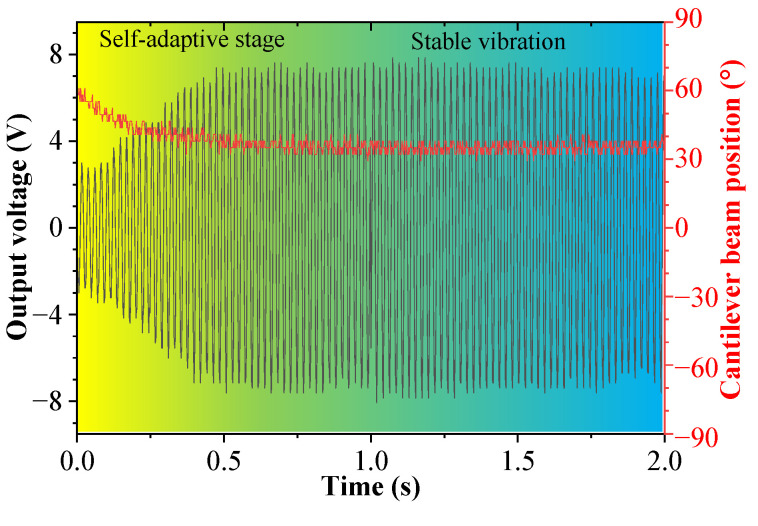
The output voltage and cantilever beam position versus time when *θ* = 60°.

Figure 10 shows the output voltage and cantilever beam’s position versus time when the rolling angle is *θ* = 60°. The red line represents the position of the cantilever beam, and the black line represents the output voltage of the piezoelectric patch. For the convenience of understanding, the position of the cantilever beam is represented by the orientation angle between its normal direction and the excitation direction; that is, when the normal direction of the cantilever beam is consistent with the excitation’s direction, the orientation angle of the cantilever beam position is 0°. It can be observed from the figure that the orientation angle of the cantilever beam position gradually decreases to the steady-state vibration position, and the output voltage amplitude of the cantilever beam increases with a decrease in the orientation angle, ultimately reaching a stable voltage amplitude. At the initial stage, the orientation angle of the cantilever beam position is 60°, and at the final steady-state vibration, the orientation angle is approximately 35°. The cantilever beam is not located at the ideal 0° position during steady-state vibrations due to the effect of the resistance moment generated by the eccentric gravity of the tip mass on the energy harvester.

**Figure 11:**
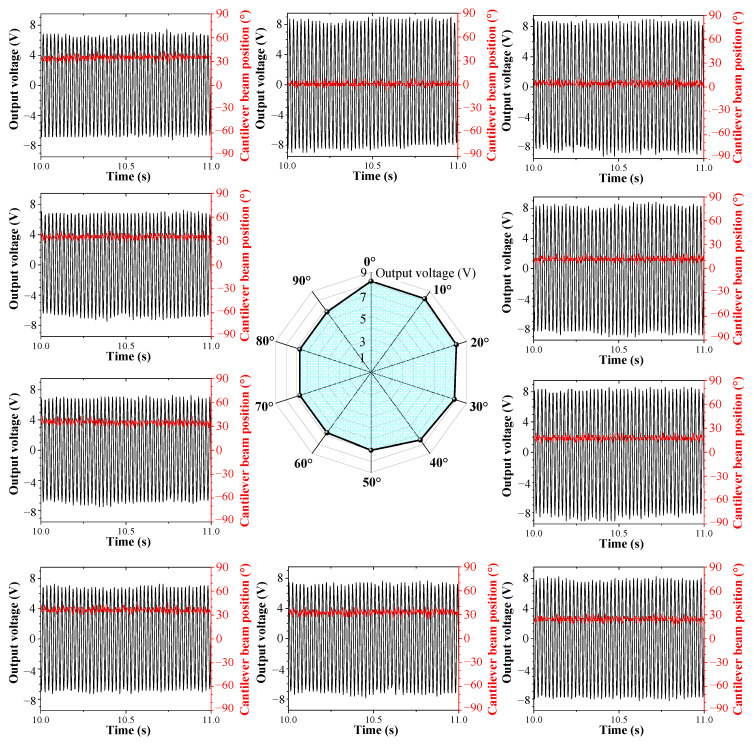
Steady-state output under different rolling angles when the mass eccentricity coefficient is *r* = 3 mm.

Figure 11 shows the steady-state vibration positions and voltage output amplitudes of the cantilever beam under different rolling angles when the mass eccentricity coefficient is *r* = 3 mm. It can be observed from the figure that different rolling angles correspond to different steady-state vibration positions, resulting in different voltage output amplitudes. On the one hand, eccentric gravity drives the cantilever beam to rotate under external excitation; on the other hand, eccentric gravity generates a resistance moment that prevents the cantilever beam from rotating. When the rolling angle of the excitation is small (such as *θ* = 10° or *θ* = 20°), the amplitude response of the cantilever beam is large, the rotational torque generated by the centrifugal force of vibrations can overcome the resistance moment of eccentric gravity, driving the cantilever beam to rotate to a position that is roughly consistent with the excitation’s direction. When the rolling angle is large (such as *θ* = 60°), the amplitude response of the cantilever beam is very small and can only drive the cantilever beam to rotate to a certain position. Figure 10 shows that the cantilever beam produces balance near an orientation angle of 35°, and as the rolling angle further increases (such as *θ* = 80°), the equilibrium position hardly changes as the resistance moment and vibration’s centrifugal force reach an equilibrium state.

**Figure 12:**
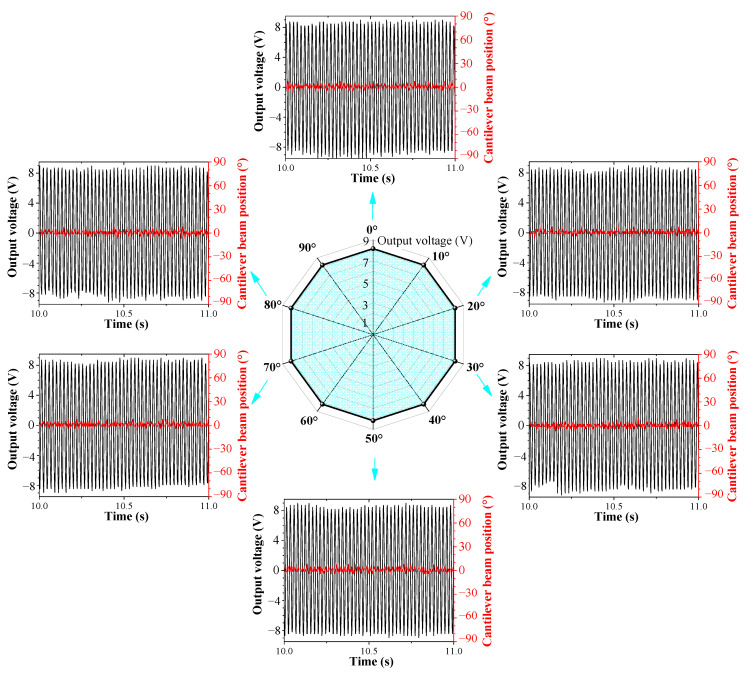
Steady-state output under different rolling angles when the mass eccentricity coefficient is *r* = 0 mm.

**Figure 13:**
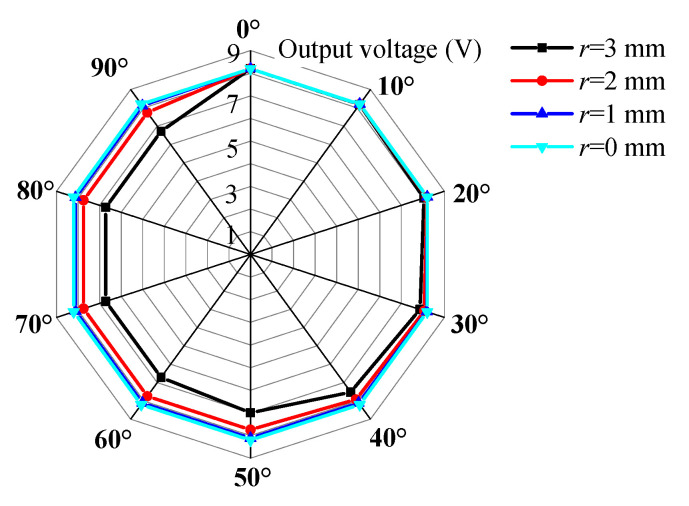
Steady-state output voltage amplitude with different mass eccentricity coefficients.

**Figure 14:**
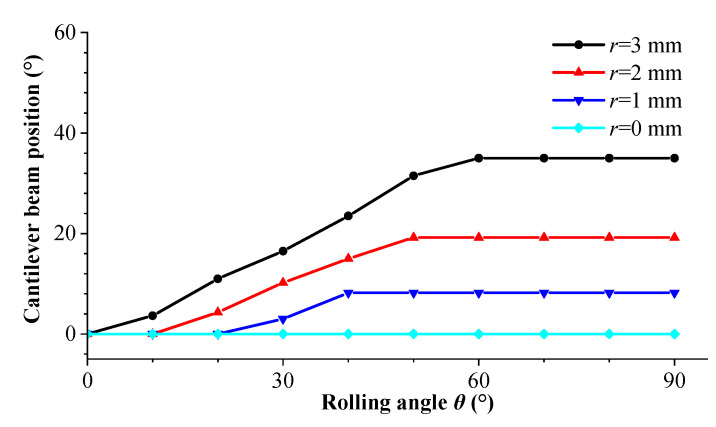
Steady-state vibration positions with different mass eccentricity coefficients.

According to Figure 11, it can be observed that due to the presence of eccentric gravity, when the rolling angle is large, the cantilever beam cannot rotate to the ideal 0° position for large-amplitude vibrations. The steady-state vibration position of the cantilever beam is related to the magnitude of mass eccentricity coefficient *r*. Figure 12 shows the steady-state vibration positions and voltage output amplitudes of the cantilever beam under different rolling angles when the mass eccentricity coefficient is *r* = 0 mm. It can be observed from the figure that due to the absence of an additional resistance moment, the cantilever beam can rotate to the 0° position for large-amplitude vibrations regardless of the change in external excitation direction. It should be noted that when the rolling angle is *θ* = 90°, theoretically, the cantilever beam cannot rotate to the 0° position, but in practice, due to unbalanced mass or other reasons caused by manufacturing and assembly processes, the cantilever beam can also rotate to the 0° position, so there is also a large voltage output from the cantilever beam in this excitation direction. Figure 13 and Figure 14 show the steady-state output voltage amplitude and steady-state vibration position under different mass eccentricity coefficients: The steady-state output voltage amplitude is related to the position of the cantilever beam. When the orientation angle of the steady-state vibration position is small, the output voltage amplitude is larger, and vice versa, the output voltage amplitude is smaller. From the figure, it can be observed that when the mass eccentricity coefficient changes from large to small, the output voltage amplitude of the cantilever beam increases, and the wrapped area within the excitation range of 0°~+90° increases. When the mass eccentricity coefficient is *r* = 0 mm, the maximum working area indicates that the energy harvester has the best directional adaptability in the two-dimensional space.

**Figure 15:**
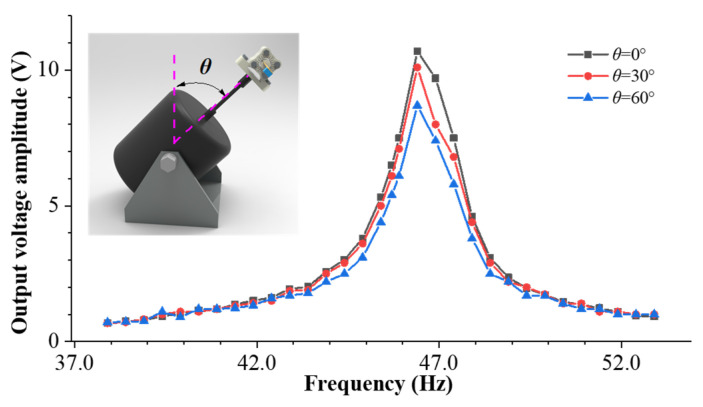
Experimental sweep frequency curve for different rolling angles when the mass eccentricity coefficient is *r* = 3 mm.

Figure 15 shows the sweep frequency curve for different rolling angles when the mass eccentricity coefficient is *r* = 3 mm. When the rolling angle changes, due to the directional self-adaptive characteristics, the cantilever beam can always rotate to a certain position where the resistance moment generated by the eccentric gravity and the rotation moment generated by the vibration centrifugal force reach a balanced torque state. Different rolling angles correspond to different steady-state vibration positions, which correspond to different output voltage amplitudes. However, the natural frequency of the cantilever beam does not change, which corresponds to Equation (2).

In summary, under actual installation angles, when the external excitation changes in the rolling direction, due to the self-adaptive feature of the cantilever beam, the energy harvesting system can automatically adjust to a certain position for stable vibration. Due to the existence of eccentric gravity, changes in the rolling angle will generate a rotational resistance moment, which affects the steady-state vibration position and voltage output amplitude of the system, but it does not affect the natural frequency of the cantilever beam.

### 5.2. The Output Characteristics of the System When the Excitation Changes in the Pitch Direction

This section analyzes the output characteristics of the energy harvesting system when the excitation direction changes in the pitch direction. Due to the pitch angle between the normal direction of the cantilever beam and the external excitation direction, the excitation acting on the cantilever beam is decomposed into two parts: one part is applied to the normal direction of the cantilever beam, driving the cantilever beam to vibrate; the other part is applied to the axis direction of the cantilever beam, generating an axial force that affects the natural frequency of the cantilever beam.

**Figure 16:**
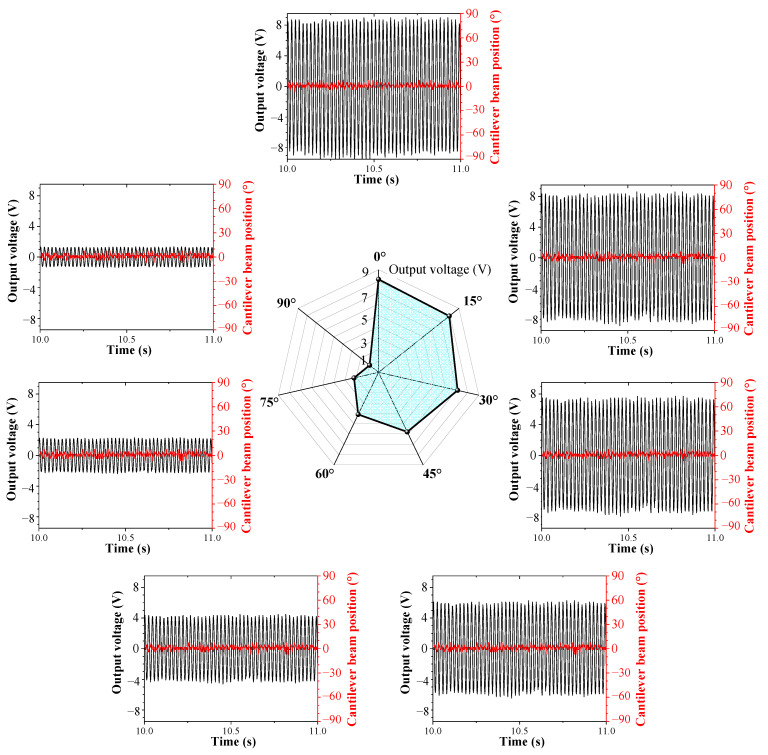
Steady-state vibration position and output voltage under different pitch angles.

**Figure 17:**
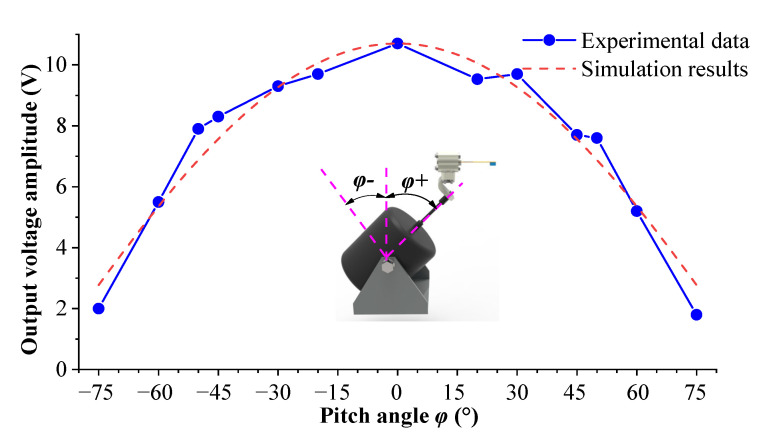
Sweep voltage under different pitch angles.

Figure 16 shows the steady-state vibration position and output voltage of the cantilever beam at different pitch angles. The red curve in the figure represents the position of the cantilever beam in the rolling direction. From the figure, it can be observed that as the pitch angle increases, the steady-state position of the cantilever beam in the rolling direction remains unchanged, but the output voltage amplitude decreases as the pitch angle increases. According to Equation (2), it can be observed that as the pitch angle increases, the component force of the external excitation in the normal direction of the cantilever beam will gradually decrease in a manner exhibiting a trigonometric function; that is, the excitation intensity of the cantilever beam will decrease, so its output voltage will also decrease. Similar results are obtained during the experimental frequency sweep process, as shown in Figure 17. From this perspective, the adaptability of the DSPEH in the pitch direction is poor, which is the same as a conventional PEH.

**Figure 18:**
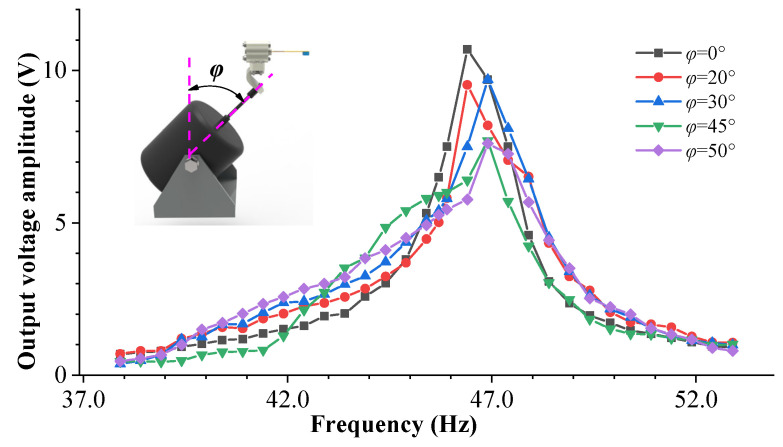
The frequency sweep curves of different pitch angles.

**Figure 19:**
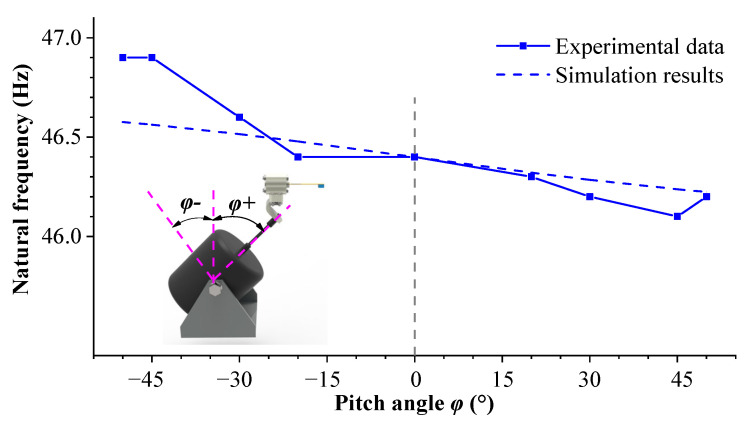
The variation curve of the natural frequency of the cantilever beam with the pitch angle.

Figure 18 shows the sweep frequency curves of the energy harvesting system under different pitch angles. It can be observed from the figure that as the pitch angle changes, the natural frequency shifts to the right and the output voltage peak decreases. The natural frequency shift in the experiment indicates that the additional axial load caused by the pitch angle has an impact on the cantilever beam. Figure 19 shows the natural frequency curves of the cantilever beam under different pitch angles. The solid line represents the experimental results, and the dashed line represents the simulation data. Due to the small excitation acceleration (0.7 g) during the experimental process, the additional axial force generated by the excitation component is also very small, so the natural frequency changes little. In addition, there are uncontrollable factors and measurement errors during the experimental process, and the natural frequencies of the experiment and simulation cannot fully correspond with one another. However, the trend of the two is the same: in the range of 0~+90°, the natural frequency of the cantilever beam decreases with an increase in the pitch angle; within the range of −90~0°, the natural frequency increases with an increase in pitch angle.

### 5.3. The Output Characteristics of the System When the Excitation Changes in Multiple Directions

**Figure 20:**
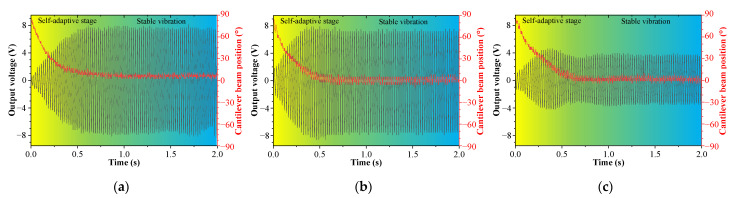
The output voltage and cantilever beam position versus time when *θ* = 90°: (**a**) *φ* = 0°; (**b**) *φ* = 30°; (**c**) *φ* = 60°.

When the external excitation changes simultaneously in pitch and rolling directions, the output voltage of the cantilever beam and the curve of the position of the cantilever beam versus time are shown in Figure 20, where Figure 20a represents *φ* = 0° and *θ*= 90°, Figure 20b represents *φ* = 30° and *θ* = 90°, and Figure 20c represents *φ* = 60° and *θ* = 90°.

For different initial rolling angles, the DSPEH can track the external excitation direction by automatically calibrating the position of the cantilever beam, ultimately reaching the same position and stabilizing the vibration, indicating that the DSPEH also has the feature of self-adaptive energy harvesting in the rolling direction under different pitch directions. Although the final steady-state position for different pitch directions is the same, the output voltage is different, because the pitch angle causes different excitation components acting on the cantilever beam, which is the same as the analysis in the previous section.

**Figure 21:**
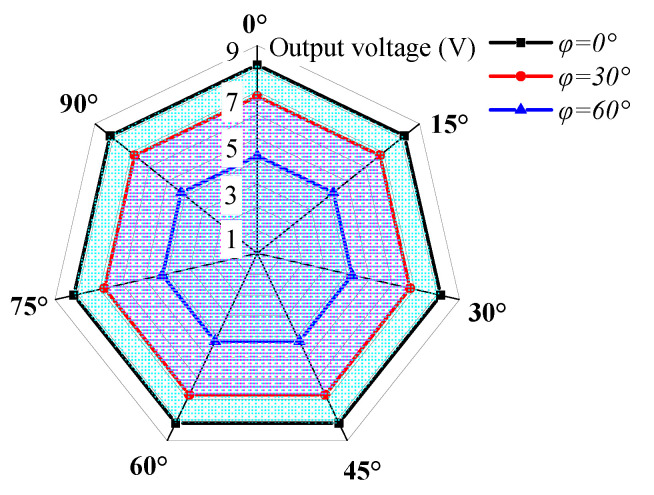
The steady-state output voltage amplitude of the DSPEH in the rolling direction.

Figure 21 shows the steady-state output voltage amplitude of the cantilever beam at different rolling angles when the pitch angles are 0°, 30°, and 60°. From the figure, it can be observed that due to the self-adaptive feature of the energy harvester in the rolling direction, the output characteristics of the harvester are the same at different rolling angles, while the output voltage varies at different pitch angles. As the pitch angle increases, the area covered by the voltage amplitude in the graph decreases.

**Figure 22:**
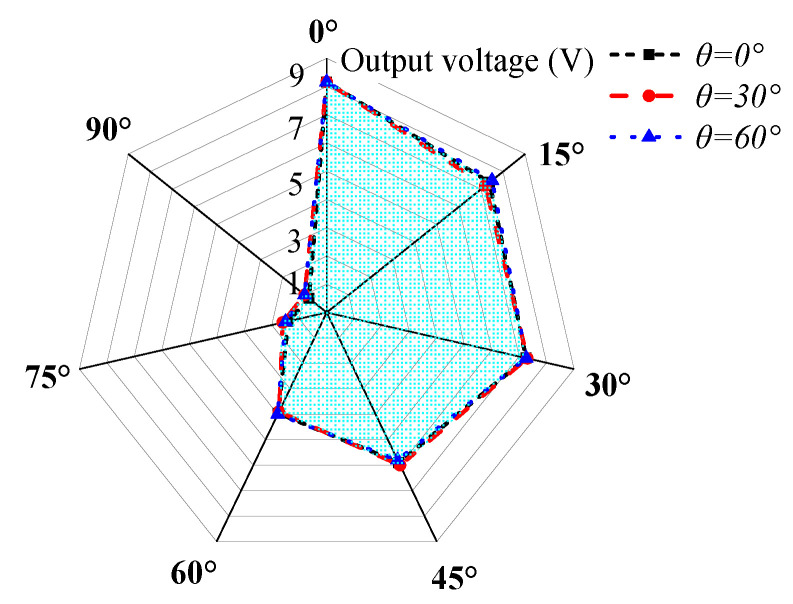
The steady-state output voltage amplitude of the DSPEH in the pitch direction.

Figure 22 shows the steady-state output voltage amplitude of the cantilever beam at different pitch angles when the rolling angles are 0°, 30°, and 60°. As observed from the figure, due to the self-adaptive feature of the energy harvester in the rolling direction, the output characteristics of the harvester are the same at different rolling angles. According to the above analysis, the main factor affecting the multi-directional performance of the energy harvester is the pitch angle, and the directional self-adaptive feature allows the harvester to fully adapt to the rolling angle.

### 5.4. Performance Analysis and Evaluation of a Multi-Directional Energy Harvester

**Figure 23:**
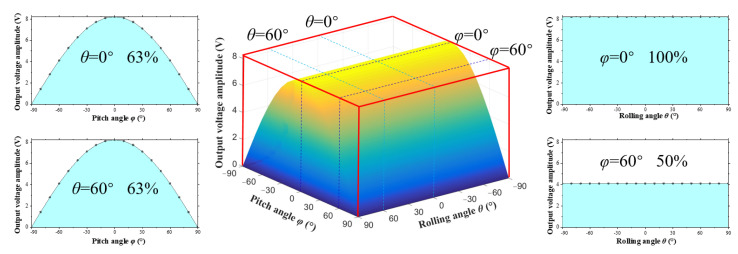
The energy harvesting workspace of the DSPEH when the mass eccentricity coefficient is *r* = 0 mm.

**Figure 24:**
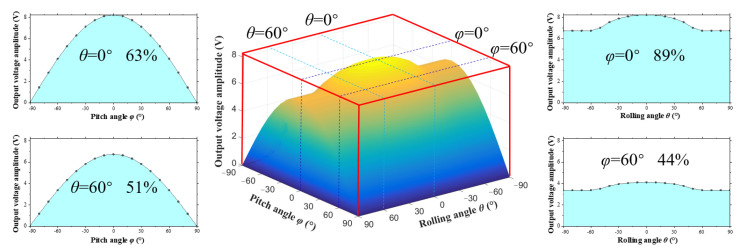
The energy harvesting workspace of the DSPEH when the mass eccentricity coefficient is *r* = 3 mm.

In summary, when the excitation direction changes in three-dimensional space, the external excitation direction has a significant impact on the output characteristics of the energy harvesting system. In order to evaluate the output performance of energy harvesters under complex excitations, the concept of “Energy Harvesting Workspace” is put forward to describe the ability of energy harvesting in multiple directions. The energy harvesting workspace is represented by the angles of external excitation and the amplitude of output voltage. In the coordinate system, the horizontal axis represents the angles of the external excitation, and the vertical axis represents the amplitude of the output voltage. The energy harvesting workspaces of the DSPEH under different mass eccentricity coefficients are shown in Figure 23 and Figure 24.

From Figure 23, it can be observed that when the mass eccentricity coefficient is *r* = 0 mm, the three-dimensional workspace of the DSPEH is arched, and its boundary is limited by the different voltage amplitudes generated by pitch angle *φ*. By converting angles to radians, the volume wrapped by the arch is 50.9 V, occupying 63% of the total three-dimensional space (80.9 V). When external excitation changes in the two-dimensional space, the two-dimensional workspace of the DSPEH is equivalent to the two-dimensional cross-section of the three-dimensional workspace. As shown on the right side of Figure 23, due to its direction self-adaptive feature, the DSPEH can always vibrate stably at a certain position, and the output voltage amplitude in the rolling direction is consistent. When the pitch angle is *φ* = 0°, the output voltage’s amplitude curve covers an area of 25.8 V within −90~+90°, occupying 100% of the total two-dimensional space (25.8 V). However, when the pitch angle is *φ* = 60 °, due to the limited excitation component acting on the cantilever beam, the area covered by the output voltage amplitude curve only accounts for 50% of the total area, which is significantly lower than that of *φ* = 0°. In addition, as shown on the left side of Figure 23, the area of the two-dimensional workspace obtained from the pitch angle cross-section accounts for 63% of the total area and remains unchanged, which is due to the self-adaptive feature in the rolling direction.

When the mass eccentricity coefficient is *r* = 3 mm, the workspace is an arched shape with a raised middle, and its boundary is affected by both pitch and rolling angles, as shown in Figure 24. The rolling angle affects the height of the arch: When the rolling angle changes from −90° to +90°, the height of the arch first increases and then decreases. The height of the arch at both ends is significantly smaller than that of *r* = 0 mm. This specially shaped arch is generated by mass eccentricity coefficient *r*, and its wrapping volume is 38.8 V, occupying 48% of the total three-dimensional space (80.9 V). This arch also correspondingly reduces the two-dimensional workspace; for example, when the pitch angle is *φ* = 0°, the two-dimensional space in the rolling direction accounts for 89% of the total space. When the rolling angle is *θ* = 60°, the two-dimensional space in the pitch direction accounts for 51% of the total space.

From this perspective, the energy harvester proposed in this paper has good adaptability in the direction of the two-dimensional space (rolling direction). In particular, when the mass eccentricity coefficient is *r* = 0 mm, it obtains all workspaces in the two-dimensional space. The total workspace in the three-dimensional direction depends entirely on the energy output of the second angle (pitch direction) as the pitch angle affects the size of the excitation component, which limits the ability of the energy harvester to work in multiple directions.

## 6. Conclusions

This paper discusses in detail the directional self-adaptation performance of the DSPEH. The rolling angle and the pitch angle are used to define complex excitations in three-dimensional space. A distributed parameter model of the energy harvesting system is established based on the moment balance and force balance equations of the microelement. The variation law of the natural frequency and rotational resistance moment of the cantilever beam when the external excitation direction changes are studied, as well as the dynamic response of the DSPEH in different excitation directions. The main conclusions are as follows. Firstly, the natural frequency of the cantilever beam is related to the pitch angle of the external excitation and is not related to the rolling angle. The resistance moment of the cantilever beam exhibits a trigonometric relationship relative to the pitch angle and the rolling angle, and it increases with an increase in the rolling angle. Secondly, the output characteristics of the DSPEH are studied when the rolling angle and pitch angle of the external excitation change individually or simultaneously. Due to the directional self-adaptive feature of the energy harvester in the rolling direction, the main factor affecting multi-directional performance is pitch angle. Thirdly, in order to describe the output performance of energy harvesting systems in multiple directions, this paper puts forward the concept of “Energy Harvesting Workspace” and establishes a performance evaluation method for multi-directional energy harvesting. The workspace is expressed using external excitation angles and output voltage amplitudes, and the output performance of energy harvesters is evaluated using volume-wrapping and area-covering methods. The DSPEH has excellent directional adaptability in the two-dimensional space (rolling direction), especially when the mass eccentricity coefficient is *r* = 0 mm, achieving 100% of the workspace in the two-dimensional space. The total workspace in the three-dimensional direction depends entirely on the energy harvesting efficiency of the pitch angle. The volume of the workspace package is 50.9 V, occupying 63% of the total space (80.9 V).

## Figures and Tables

**Table 1:** Parametric values of the DSPEH.

Cantilever Beam		Piezoelectric Patch	
Parameters	Values	Parameters	Values
Material	Brass	Material	PVDF
Density (10^3^ Kg/m^3^)	8.6	Length *L_p_* (m)	0.020
Length *L* (m)	0.052	Width *W_p_* (m)	0.008
Width *W* (m)	0.008	Thickness *H_p_* (m)	0.000028
Thickness *H* (m)	0.0006	Equivalent electromechanical coupling coefficient *α* (10^−5^ Cm^−1^)	−7.333
Elastic modulus *E* (GPa)	120	Equivalent capacitance *C_P_* (10^−9^ F)	6.25
Damping coefficient *c*_1_ (Ns/m)	0.1	Equivalent resistance of the energy harvesting circuit *R* (10^6^Ω)	7.0
**Tip Mass**		**Rotation Unit**	
**Parameters**	**Values**	**Parameters**	**Values**
Material	Stainless steel	Support shaft material	Polyurethane
Weight (Kg)	0.0031	Deep Groove Ball Bearing model	688
Gravity acceleration *g* (m/s^2^)	9.8	Damping coefficient *c*_2_ (Ns)	0.00002
Mass eccentricity distance *r* (m)	0.003	Friction torque (Nm)	0

## Data Availability

Not applicable.
